# Supplementary material for: SyNDI: synchronous network data integration framework
Source: BMC Bioinformatics. 2018 Nov 6;19:403. doi: 10.1186/s12859-018-2426-5 (PMC6219086; doi:10.1186/s12859-018-2426-5)
Supplement: Supplementary file 5 — Files of the example presented in Additional file 4. This additional file is a zip package that contains all files used in Additional file 4. Detailed descriptions of each of these files are presented in a README file included in this zip package. (GZ 30411 kb) [file 12859_2018_2426_MOESM5_ESM.gz › SyNDITools/Meme2Fimo/meme_4.9.0_4.tar.gz!/meme_4.9.0/doc/scpd2meme.html]

scpd2meme


# scpd2meme

**Usage:** `scpd2meme [options] <matrix file>`

**Description:**

Convert an SCPD matrix file into a MEME version 4 formatted file
suitable for use with MAST and other MEME Suite programs.

**Input:**

An SCPD matrix file describes one ore more motifs in terms of letter
frequencies.

A probability matrix and optionally a log-odds matrix are output for each
motif in the file. The probability matrix is computed using
pseudo-counts consisting of the background frequency (see -bg, below)
multiplied by the total pseudocounts (see -pseudo, below). The log-odds
matrix uses the background frequencies in the denominator and is log
base 2.

**Options:**

-skip <scpd ID>
:   skip this ID (may be repeated)

-numbers
:   use numbers instead of strings as motif names

-bg <background file>
:   file with background frequencies of letters; default: uniform background

-pseudo <total pseudocounts>
:   add <total pseudocounts> times letter background to each frequency; default: 0

-logodds
:   print log-odds matrix, too; default: print frequency matrix only

-url <website>
:   website for the motif; The scpd ID is substituted for MOTIF\_NAME; default: no url

**Output:**

Writes MEME format to standard output.

**Sample Input:**

```
>RLM1   22      524     MCB 1997, 17:1848-1859
A  10  14  11   0   0   0  42  16  22  23  32   0  42   0  27   4   7  10
T   8  10  30  37   0  42   0  26  20  19  10  42   0   0   1  20  16   7
G  10  16   1   5   0   0   0   0   0   0   0   0   0  42   0   2   3  10
C  10   2   0   0  42   0   0   0   0   0   0   0   0   0  14  15  16  13
>SMP1   11      405     MCB 1997, 17:1848-1859
A   8   8  11   0   0   8   0   0  30   6  13  14  21   0  30   2   8  27   9   6
T   4  12   0   9  30  11   1  30   0  24  16  16   9  30   0   0   7   1   9   7
G   5   4  19   0   0  11   0   0   0   0   0   0   0   0   0  28   0   0   7   4
C   3   5   0  21   0   0  29   0   0   0   0   0   0   0   0   0  14   0   4   9
```
